# In Vitro and Ex Vivo Antifungal Activities of Metconazole against the Rice Blast Fungus *Pyricularia oryzae*

**DOI:** 10.3390/molecules29061353

**Published:** 2024-03-19

**Authors:** Liwang Fei, Lingyun Hao

**Affiliations:** 1 Center for Plant Environmental Sensing, College of Life Sciences and Oceanography, Shenzhen University, Shenzhen 518060, China; liwang_fei@szu.edu.cn; 2 College of Physics and Optoelectronic Engineering, Shenzhen University, Shenzhen 518060, China; 3 Shenzhen Key Laboratory of Marine Bioresource & Eco-Environmental Science, Shenzhen Engineering Laboratory for Marine Algal Biotechnology, College of Life Sciences and Oceanography, Shenzhen University, Shenzhen 518060, China

**Keywords:** *Pyricularia oryzae*, rice blast, metconazole, antifungal activity, fungicide

## Abstract

Rice blast, caused by the filamentous fungus *Pyricularia oryzae*, has long been one of the major threats to almost all rice-growing areas worldwide. Metconazole, 5-(4-chlorobenzyl)-2, 2-dimethyl-1-(1H-1, 2, 4-triazol-1-ylmethyl) cyclopentanol, is a lipophilic, highly active triazole fungicide that has been applied in the control of various fungal pathogens of crops (cereals, barley, wheat), such as the *Fusarium* and *Alternaria* species. However, the antifungal activity of metconazole against *P. oryzae* is unknown. In this study, metconazole exhibited broad spectrum antifungal activities against seven *P. oryzae* strains collected from rice paddy fields and the wild type strain P131. Scanning electron microscopic analysis and fluorescein diacetate staining assays revealed that metconazole treatment damaged the cell wall integrity, cell membrane permeability and even cell viability of *P. oryzae*, resulting in deformed and shrunken hyphae. The supplementation of metconazole in vitro increased fungal sensitivity to different stresses, such as sodium dodecyl sulfate, congo red, sodium chloride, sorbitol and oxidative stress (H_2_O_2_). Metconazole could inhibit key virulence processes of *P. oryzae*, including conidial germination, germ tube elongation and appressorium formation. Furthermore, this chemical prevented *P. oryzae* from infecting barley epidermal cells by disturbing appressorium penetration and subsequent invasive hyphae development. Pathogenicity assays indicated a reduction of over 75% in the length of blast lesions in both barley and rice leaves when 10 μg/mL of metconazole was applied. This study provides evidence to understand the antifungal effects of metconazole against *P. oryzae* and demonstrates its potential in rice blast management.

## 1. Introduction

Rice (*Oryza sativa*), a major crop that feeds more than half of the world’s population, has been threatened by various plant fungal diseases [1]. One of the most concerning pathogens, *Pyricularia oryzae*, causes rice blast, which is devastating to all rice-growing areas worldwide. The disease results in 10–30% annual yield losses, which are able to feed about 60 million people in the world [2,3,4]. *P. oryzae* can infect rice plants at any developmental stage, including at the roots, stems, leaf nodes and panicles, which makes disease control rather difficult and costly [5]. In addition to rice, *P. oryzae* can also infect barley, sorghum, wheat, maize and other cultivated or non-cultivated grasses, which can all serve as reservoirs of the fungus and thus further add challenges to rice blast control [1,6,7,8,9]. Therefore, continuous efforts in the development of effective and sustainable approaches to managing rice blast are still urgently needed.

Currently, disease management strategies involve a combination of methods, including breeding resistant varieties, the application of chemical fungicides and the use of biological control agents. However, many resistant varieties tend to lose resistance after only 2–4 years of plantation due to the extremely rich genetic diversity of *P. oryzae* isolates in paddy fields and the quick emergence of novel virulent races due to genetic mutation [10,11]. Although the biological control approaches using beneficial microorganisms and their derived natural products have advantages such as lower cost and being more environmentally compatible than chemical control, their effectiveness and persistence are still insufficient after large-scale trials in fields [12]. Therefore, the use of chemical fungicides is still the primary strategy for controlling rice blast, and the most frequently used chemicals at present include tricyclazole, pyroquilon, azoxystrobin, isoprothiolane, carbendazim, pyraclostrobin, metominostrobin, tebuconazole and propiconazole [13,14,15,16,17,18]. However, the risk of resistance development and decreasing efficacy has limited the application of certain fungicides [19,20,21,22,23]. Thus, it is urgent to discover novel fungicides with high efficiency and target specificity to replace or be applied in alternation with those that are currently being used to achieve better rice blast management results.

Metconazole, a demethylation inhibitor (DMI) targeting the fungal ergosterol biosynthesis pathway, was discovered in 1986 [24]. Metconazole has been registered in France (1993), the United States (2007) and Canada (2010). In China, metconazole has been registered by Adama HuiFeng (Jiangsu) Co., Ltd. and Shanghai High Victory Fine Chemical Co., Ltd. for the control of various fungal diseases of crops (wheat and barley), such as stripe rust and dwarf rust (Agrochemical Information Net, http://www.jsppa.com.cn/, accessed on 8 February 2023). The antifungal activities of metconazole against several plant pathogens have been reported, including on *Fusarium pseudograminearum*, *F. culmorum*, *F. graminearum*, *F. verticillioides*, *Corynespora cassiicola* [25,26,27,28,29,30]. As for *C. cassiicola*, the mean EC_50_ value of metconazole for 121 *C. cassiicola* isolates was 0.78 ± 0.41 μg/mL, and the application of metconazole significantly reduced the disease incidence of *Corynespora* leaf spot in the field [28]. Moreover, metconazole effectively inhibited mycelial growth and conidial germ tube elongation in *F. pseudograminearum* at 0.05 μg/mL and disturbed the synthesis of ergosterol and significantly reduced crown rot occurrence in wheat [30]. However, the antifungal mechanism of metconazole on *P. oryzae* and its control efficacy on rice blast are still unclear. 

Once in contact with a hydrophobic surface such as rice leaves, the conidium of *P. oryzae* germinates quickly within 2 h to generate a germ tube under high humidity. Then, a specialized infection structure called appressorium forms at the end of the germ tube. During maturation, enormous turgor pressure is generated in appressorium, which produces sufficient physical force for the pathogen to successfully penetrate the host cuticle, invade and expand within rice tissues and finally form typical lesions [1]. Each of these infection processes is indispensable for successful disease development. To investigate the inhibitory effects of metconazole on *P. oryzae* development and virulence, the present study examined the effects of metconazole on mycelia growth, as well as on cell wall integrity, conidial germination and appressorium formation. Furthermore, the efficacy of metconazole against rice blast was determined by an examination of *P. oryzae* virulence in barley epidermal cells and in detached barley and rice leaves. These results provide useful information for metconazole application in rice blast management in the future. 

## 2. Results 

### 2.1. Sensitivity of P. oryzae to Metconazole

**Figure 1:**
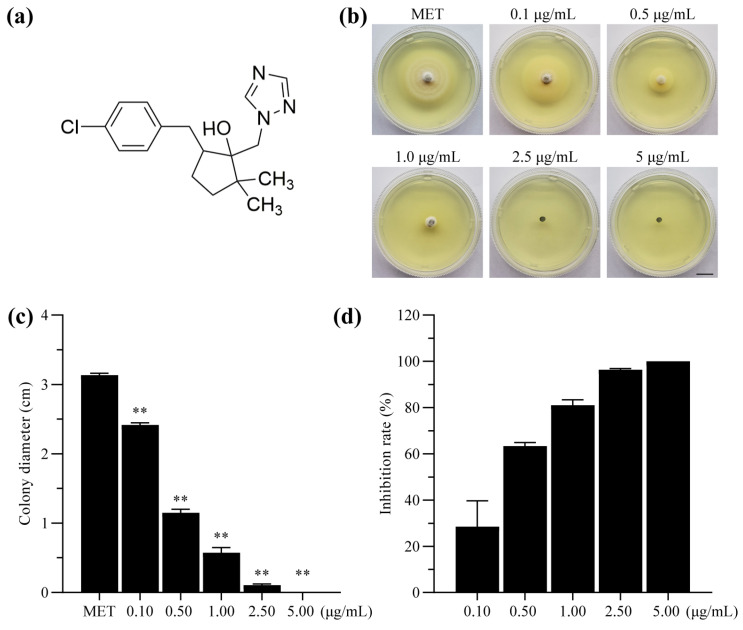
Inhibitory effects of metconazole on the mycelial growth of the *P. oryzae* strain P131. (**a**) Chemical structure of metconazole. (**b**) Colony morphology of *P. oryzae* strain P131 grown on CM plate for 5 d (Bar: 1 cm). The statistics of (**c**) colony diameter and (**d**) inhibition rates of metconazole on P131 growth. MET: methanol as solvent control, **: *p* < 0.01.

Metconazole significantly inhibited mycelial growth in *P. oryzae* strain P131 in a dose-dependent manner (Figure 1). The supplementation of 0.1 μg/mL of metconazole into the medium resulted in an approximately 28.46% inhibition rate on fungal growth, while a concentration of 0.5 μg/mL resulted in more than 60% inhibition. When the concentration of metconazole reached 2.5 μg/mL, fungal growth was almost completely inhibited. To investigate whether there is variation in sensitivity to this chemical among different *P. oryzae* isolates, seven isolates collected from the rice paddy fields in Shaoguan city were incubated on complete medium (CM) plates supplemented with metconazole of 0.5 and 1.0 μg/mL, respectively. The results showed that the inhibition rates of metconazole on all *P. oryzae* strains reached more than 80% when applied at 1.0 μg/mL (Figure S1). These results indicated that metconazole displayed strong broad-spectrum inhibitory effects on the mycelial growth of *P. oryzae*.

### 2.2. Metconazole Treatment Damaged the Cell Wall Integrity and Cell Membrane Permeability of P. oryzae

**Figure 2:**
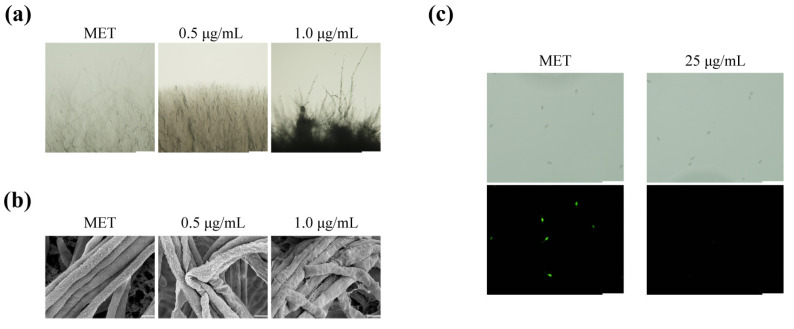
Microscopic observation of the antifungal effects of metconazole on the *P. oryzae* strain P131 (**a**) Bright field (Bar: 200 μm) and (**b**) SEM images (Bar: 2 μm) of hyphal structures in *P. oryzae* strain P131 on CM plates. (**c**) FDA staining of *P. oryzae* conidia incubated on hydrophobic glasses at 2 hpi (Bar: 100 μm). MET: methanol as solvent control.

To further understand how metconazole affects the mycelial growth of *P. oryzae*, the morphology of the mycelia sampled from the sensitivity assays of *P. oryzae* to metconazole in Section 2.1 was observed under a microscope (BX51, Olympus Corporation, Tokyo, Japan). The edge of stressed *P. oryzae* hyphae became more compacted with an increase in the concentration of metconazole (Figure 2a). The SEM images showed that the untreated hyphae of P131 appeared to be smooth and fluffy. In contrast, fungal hyphae treated with 1 μg/mL metconazole became rough, shrunken, deformed and twisted (Figure 2b). Subsequent FDA staining assay demonstrated that no green fluorescence was observed in the treated samples, while strong fluorescence was observed in the untreated ones, suggesting that metconazole treatment might affect the cell membrane permeability and cell viability of conidia in *P. oryzae* (Figure 2c). 

**Figure 3:**
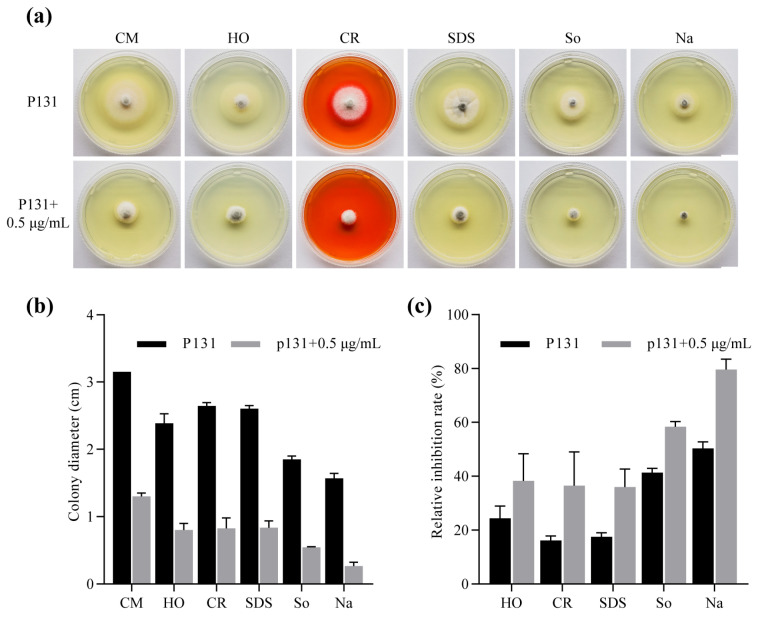
Increased sensitivity of *P. oryzae* in response to various stress upon 0.5 μg/mL metconazole treatment. (**a**) Colony morphology of indicated strains grown on CM plates (Bar: 1 cm). The statistics of (**b**) colony diameter and (**c**) inhibition rates of indicated strains grown on CM plates supplemented with different stresses. CM: as a medium control, HO: 10 mM H_2_O_2_, CR: 0.2 mg/mL Congo red, SDS: 0.005% Sodium dodecyl sulfate, So: 1 M Sorbitol, Na: 0.7 M NaCl.

To determine the impacts of metconazole on P131 in response to various environmental stresses during fungal development, fungal growth with or without the supplementation of metconazole under different stress conditions was determined. As shown in Figure 3, compared with the untreated strains, the treated strain was more sensitive to cell wall inhibitors, including 0.005% sodium dodecyl sulfate (SDS) and 0.2 mg/mL congo red (CR), which indicated that metconazole treatment destroyed the cell wall’s integrity. Furthermore, the treated strain was more sensitive to high osmotic pressure (1 M sorbitol and 0.7 M NaCl) and 10 mM H_2_O_2_, suggesting that metconazole treatment disturbed the permeability of the cell membrane, which was consistent with the FDA staining results. Taken together, the results of fungal growth under stressed conditions were consistent with those from the microscopic observations, both of which suggested that metconazole inhibited mycelial growth by damaging cell wall integrity and cell membrane permeability.

### 2.3. The Effects of Metconazole on P131 Conidial Germination and Germ Tube Elongation

**Figure 4:**
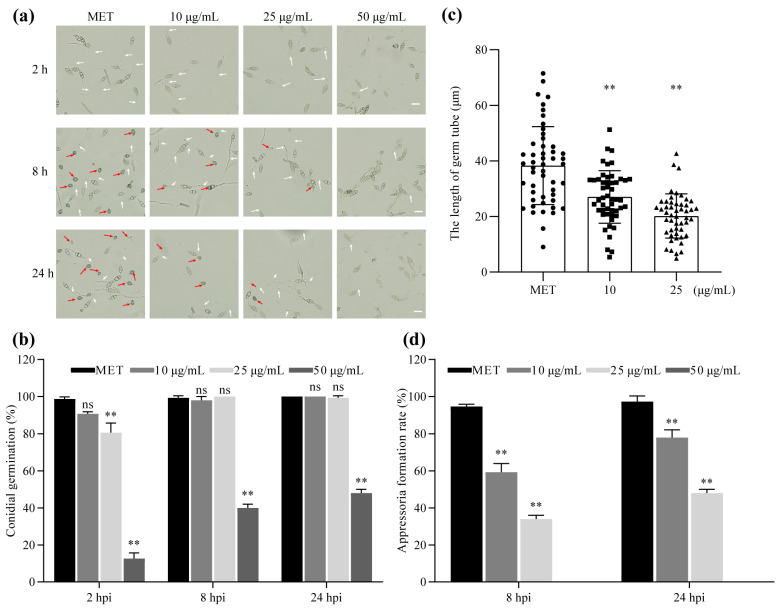
Inhibitory effects of metconazole treatment on conidial germination, germ tube elongation and appressorium formation of *P. oryzae*. (**a**) Bright field images of *P. oryzae* strain P131 conidia treated with different concentrations of metconazole at different time points. White arrows indicate germ tubes, red arrows indicate appressoria (Bar: 20 μm). (**b**) Percentages of conidial germination at 2, 8 and 24 hpi, (**c**) Average length of germ tube at 2 hpi. (**d**) The appressorium formation rates at 8 and 24 hpi. MET: methanol as solvent control, ns: *p* > 0.05, **: *p* < 0.01.

To investigate the effects of metconazole on conidial germination and germ tube elongation in the strain P131, conidial suspension (1 × 10^5^ conidia/mL) treated with or without metconazole was incubated on hydrophobic glasses for 2, 8 and 24 h, respectively. The conidial germination rates of P131 treated with 25 and 50 μg/mL of metconazole after 2 h were 80.7 and 12.7%, respectively, both of which were significantly lower than those of the untreated strain. Especially in the 50 μg/mL treatment group, the average conidial germination rate was still less than 50% at 24 h post-incubation (hpi), while the germination rate in the control group reached nearly 100% (Figure 4a,b). 

We also analyzed the lengths of germ tubes at 2 hpi using the software ImageJ v1 (National Institutes of Health, Bethesda, MD, USA). According to Figure 4c, the average length of germ tubes in P131 treated with 10 and 25 μg/mL of metconazole was 27.03 and 20.18 μm, respectively, which was significantly lower than that of the control group (38.33 μm). These results indicated that metconazole treatment significantly inhibited both conidial germination and germ tube elongation. 

### 2.4. Metconazole Treatment Hindered Appressorium Formation, Penetration and Invasive Hyphal Development of P131

Considering that metconazole treatment inhibited conidial germination and germ tube elongation, we hypothesized that metconazole affected subsequent infection steps, and thus we examined the impacts of this chemical on appressorium formation, penetration and invasive hyphal development stepwise. According to Figure 4d, only a small portion (34.00%) of P131 conidia formed immature appressoria at the tip of the germ tube when treated with 25 μg/mL of metconazole at 8 hpi, while more than 90% of the untreated conidia had formed appressoria. At 24 hpi, the average appressorium formation rate of P131 treated with the same concentration of metconazole was just 48.00%, while that of the control had reached approximately 97.33%. When 50 μg/mL of metconazole was applied, no appressorium formation was observed at both time points. 

**Figure 5:**
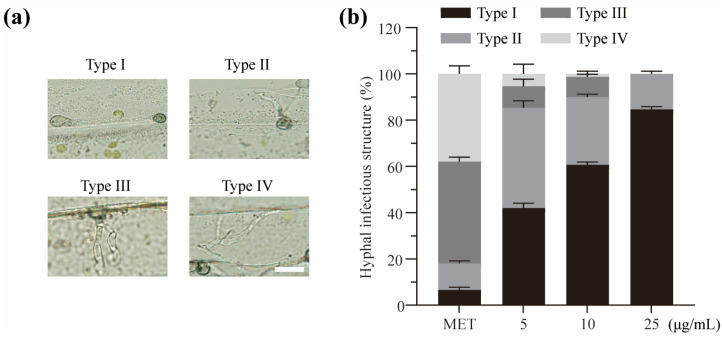
Metconazole treatment prevented *P. oryzae* from infecting barley epidermal cells. (**a**) Representative images of different types of invasive hyphae chosen from different metconazole treatments (Bar: 20 μm). (**b**) Quantitative analysis of invasive growth in barley leaf. Mean percentage of invasive hyphal growth was calculated based on an analysis of 100 appressorium penetration sites. Hyphal growth was categorized from level I to level IV (I, no penetration; II, with primary invasive hyphae; III, secondary invasive hyphae does not expand to the neighboring plant cells; IV, invasive hyphae expanding into neighboring plant cells). MET: methanol as solvent control.

Next, we examined the impacts of metconazole treatment on the appressorial penetration (AP) and invasive hyphal (IH) growth of P131 in barley epidermis. The hyphal growth was examined at 32 hpi and categorized as level I to level IV (I, no penetration; II, with primary invasion hyphae; III, with secondary invasive hyphae which does not expand to neighboring plant cells; IV, with invasion hyphae extended into neighboring plant cells, Figure 5a). As shown in Figure 5b, compared with the untreated strain, the treated strains were severely impaired in developing IH in host cells. In the untreated strain, 82% of penetration sites showed level III and IV invasive growth. In contrast, more than 84% of penetration sites showed only level I and level II growth, and less than 16% showed level III and IV growth when treated with 5 μg/mL metconazole. When treated with 10 and 25 μg/mL metconazole, more than 90% of penetration sites showed only level I and level II invasive growth. Taken together, these results suggested metconazole treatment inhibited the appressorium formation of *P. oryzae* in a dose-dependent manner and significantly blocked the AP formation and expansion of IH in the host cells.

### 2.5. Metconazole Treatment Destroyed Full Virulence of P. oryzae

**Figure 6:**
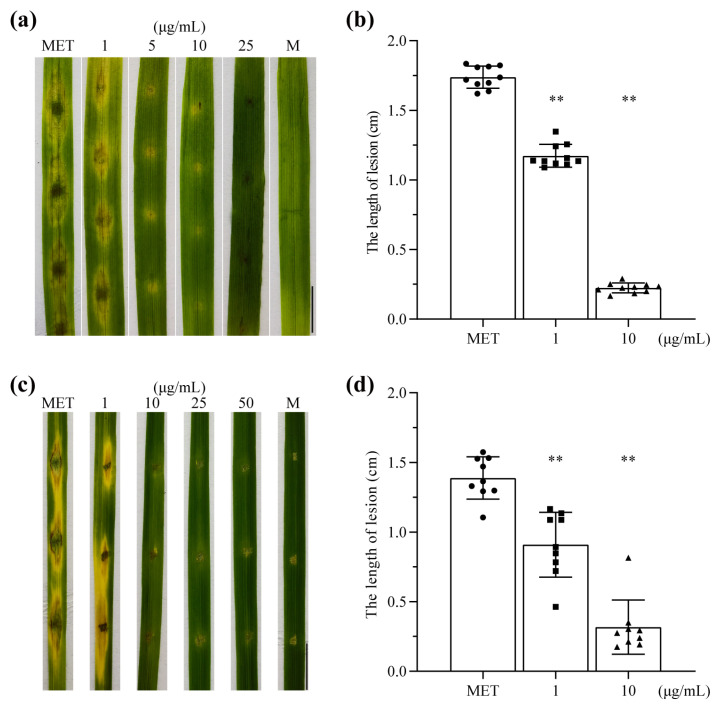
Metconazole treatment damaged the full virulence of *P. oryzae* in barley leaves and rice leaves. (**a**) The lesion image of lesion in barley leaves (Bar: 1 cm). (**b**) The statistics of the length of lesion in barley leaves. (**c**) The lesion image of lesion in rice leaves (Bar: 1 cm). (**d**) The statistics of the length of lesion in rice leaves. MET: methanol as solvent control, M: mock, incubated with distilled water amended with 0.025% tween 20, as negative control, **: *p* < 0.01.

To determine the impacts of metconazole on *P. oryzae* strain P131 virulence, we inoculated abraded rice and barley leaves with P131 conidial suspension and compared disease development with and without metconazole treatment. At 5 days post inoculation (dpi), the untreated strain formed typical fusiform disease lesions with large yellowing areas around the wounded sites in barley leaves, while the treated strain produced smaller lesions with decreased lengths as the concentration of metconazole increased (Figure 6a,b). When 10 μg/mL of metconazole was applied, the average length of the lesion was 0.224 cm, which was significantly lower than that of the control group (1.738 cm, *p* < 0.01). When treated with 25 μg/mL of metconazole or above, symptom development was completely blocked, and the morphology of wounded sites was similar to the negative control. Similar results were observed in rice leaves, namely that only minor lesions were formed with little surrounding discoloration when leaves were treated with metconazole of 10 μg/mL, and the average length of lesions was less than that of the control (Figure 6c,d). These results indicate that metconazole treatment could damage the full virulence of *P. oryzae* in host issues.

## 3. Discussion

Rice blast, caused by *P. oryzae*, is one of the most devastating fungal diseases throughout the world and leads to vital losses in major rice production regions worldwide every year [1,2,4]. At present, fungicide application is the primary strategy for disease control. The most widely used commercial fungicides for rice blast mainly include Azoxystrobin in Quadris^TM^, Syngentae (9% of the global fungicide market in China), Tricyclazole in Segard^TM^, DowAgrosciences^d^, Probenazole in Oryzemate^TM^, Meiji Seika^c^ and Propiconazole in Tilt^TM^, Syngenta^e^ [31]. The action mechanisms of these chemical fungicides mainly focus on the process relative to the virulence of *P. oryzae*. For example, tricyclazole specifically inhibits the reduction steps (polyhydroxyl-naphthalene reductase) in the DHN-melanin biosynthesis pathway and thus influences the efficiency of appressorial penetration into the host [32,33]. In addition, chitosan, a polysaccharide, was reported to damage cell wall and cell membrane integrity and inhibit septin-mediated rice plant infection [34]. Interestingly, herbicides, including metazachlor, cafenstrole and diallate, showed strong effects on very-long-chain fatty acid (VLCFA) biosynthesis as well as disturbing septin-mediated rice plant infection [35]. However, the risk of resistance development, decreasing control efficacy and environmental security have limited fungicide longevity for the control of rice blast. For example, resistance to carpropamid (a melanin biosynthetic inhibitors) was reported in Japan in 2001 [22]. Resistance to carbendazim (a benzimidazole fungicide) was also reported in China in 2004 [23]. Therefore, it is necessary to explore new chemicals with good efficacy to expand existing choices for rice blast control.

DMI fungicides are the largest group of antifungal compounds that are widely used in modern agriculture and have been utilized in the control of plant diseases caused by *Ascomycetes*, *Basidiomycetes* and Fungi imperfecti. Epoxiconazole and propiconazole, belonging to the DMI fungicide group, have been reported to effectively control rice blast [36,37,38]. Metconazole is a DMI fungicide, and previous studies have shown that this fungicide has strong inhibitory effects on the mycelial growth of *F. graminearum* and *F. pseudograminearum* [30,39,40]. Our results were similar to these findings and demonstrated that metconazole treatment led to approximately 81.08% inhibition on *P. oryzae* mycelial growth at 1 μg/mL (Figure 1). Furthermore, metconazole demonstrated broad-spectrum activity against several *P. oryzae* isolates from paddy fields with varied efficacy. To the best of our knowledge, our study is the first to demonstrate that metconazole has strong antifungal bioactivity against multiple isolates of *P. oryzae*.

In vitro metconazole treatment led to a series of changes in *P. oryzae* mycelial morphology, such as deformed and shrunken hyphae observed by SEM analysis. FDA staining results further suggested the chemical disturbed fungal cell viability of conidia (Figure 2). Fluorescein diacetate is a non-fluorescent and non-polar dye that can penetrate an intact cell membrane and can subsequently be converted into polar and green luciferin by intracellular esterase in living cells, while fluorescein diacetate cannot be decomposed in dead cells. Therefore, the strong fluorescence observed in the control group suggested high cell viability and intact cell membrane, while such a phenomenon disappeared in the metconazole-treated group, suggesting a functional cell membrane was damaged and the cell had low viability or was even completely dead. The cell wall and membrane are the outermost physical layers that protect *P. oryzae* from environmental stresses, and the disruption of these structures would lead to a failure of normal growth under stressed conditions. As expected, metconazole treatment led to the increased sensitivity of *P. oryzae* to all tested stress conditions, such as the cell wall inhibitors (sodium dodecyl sulfate and congo red), high osmotic pressure (0.7 M NaCl and 1 M sorbitol) and oxidative stress (Figure 3). Taken together, these results demonstrated that metconazole affected the cell wall integrity and altered the cell membrane permeability of *P. oryzae*. Previous reports have shown that DMI fungicides target cytochrome P450 and disturb fungal ergosterol biosynthesis, which reduces the ergosterol that is the main component of the fungal cell plasma membrane and damages the cell membrane’s function [30,41,42]. Therefore, further experiments, such as an examination of the ergosterol production and expression of genes (such as *MoErg4* [43], *MoCyp521A* and *MoCyp521B* [44]) related to ergosterol biosynthesisin *P. oryzae* treated with metconazole and determination of action sites of metconazole should be conducted to clarify the mechanisms involved in the morphological changes upon chemical treatment.

The phenotypic characterization of key virulence structures of *P. oryzae* suggested that metconazole primarily inhibited conidial germination, hindered the formation of appressorium in vitro (Figure 4) and suppressed subsequent penetration and invasive hyphae development in barley epidermal cells (Figure 5). Comparable to other pathogens such as *Fusarium* sp., the efficacy of the chemical on conidial germination rate and germ tube elongation in *P. oryzae* were similar between the two pathogens under similar concentrations [30]. All above results indicated that besides possibly targeting the biosynthetic pathway of ergosterol to damage cell membrane integrity and permeability, metconazole might also act on the pathways related to appressorium maturation and invasive hyphal development to destroy the full virulence of *P. oryzae*. Next, further experiments using a combination of approaches including RNA-seq, qRT-PCR and gene knockout analysis should be applied to screen new targets of metconazole in *P. oryzae*, which would be useful to facilitate the development of novel fungicides. Furthermore, the virulence assays showed that metconazole significantly suppressed disease development in barley and rice leaves (Figure 6). These results suggested great potential in metconazole for rice blast management.

In conclusion, our study was the first to demonstrate that metconazole significantly inhibited *P. oryzae* mycelial growth and key infection steps, including conidial germination, appressorium formation and invasive hyphae expansion, leading to strong disease suppression. A further examination of its efficacy in disease control under greenhouse and field conditions and an investigation of the molecular mechanisms involved in ergosterol biosynthesis and cellular energy metabolic pathways will facilitate the application of metconazole in rice blast management.

## 4. Materials and Methods

### 4.1. Fungicide and Pyricularia oryzae Isolates

Technical-grade metconazole (M115373) was purchased from Shanghai Aladdin Biochemical Technology Corporation, Shanghai, China. Metconazole was dissolved in methanol to make a 50 mg/mL stock solution and then kept at −20 °C for further fungicide sensitivity tests. Seven *P. oryzae* strains isolated from Guangdong province in a previous study [45] and a wild type (WT) strain, P131, kindly provided by Dr. Youliang Peng from China Agricultural University (CAU), were used in fungicide sensitivity assays. All above strains were incubated on CM plates in an illumination incubator at 28 °C [44].

### 4.2. Sensitivity of P. oryzae Isolates to Metconazole

To evaluate the sensitivity of *P. oryzae* strain P131 to metconazole, mycelial plugs of 0.3 cm in diameter taken from the edge of a 7-day-old colony were inoculated on CM plates supplemented with metconazole at a series of concentrations (0.1, 0.5, 1.0, 2.5 and 5.0 μg/mL) and cultured in an illumination incubator (photoperiod of 12 h light/12 h dark and at 28 °C) for 5 d. Plates supplemented with 0.1% methanol were used as solvent control. To test the metconazole resistance of *P. oryzae* strains isolated from paddy fields in Shaoguan city, Guangdong Province, mycelial plugs of seven isolates were inoculated on CM plates supplemented with 0.5 and 1.0 μg/mL of metconazole and cultured at 28 °C for 5 d.

To examine the impacts of metconazole on fungal response to environmental stresses, *P. oryzae* strain P131 with or without 0.5 μg/mL metconazole was inoculated onto the CM plates supplemented with various stresses, including cell wall inhibitors (0.005% SDS and 0.2 mg/mL CR), high osmotic pressure (0.7 M NaCl and 1 M sorbitol) and oxidative stress (10 mM H_2_O_2_). The colony diameters were measured after 5 days post-inoculation (dpi).

All sensitivity assays were repeated three times with three replicates each time. The relative inhibition rates of mycelia growth were calculated based on colony diameter measurement using the formula as follows: relative inhibition rate (%) = (average diameter of the control—average diameter of the compound treated strain)/(average diameter of the control—diameter of the mycelia block (0.3 cm)) × 100%.

### 4.3. Scanning Electron Microscopy (SEM) and Fluorescein Diacetate (FDA) Staining

To observe the effect of metconazole on the fungal morphology of *P. oryzae*, mycelia collected from the colonies grown as described in Section 4.2 were fixed with 2.5% (*v*/*v*) glutaraldehyde solution and washed three times with phosphate buffer solution (PBS, 0.05 M, pH 7.0, Shanghai Aladdin Biochemical Technology Co., Ltd.) for 20 min each. Then, the samples were dehydrated with a series of ethanol solutions (30, 50, 75, 90 and 95%) for 15 min, followed by 100% for 20 min in absolute ethyl alcohol. Finally, the samples were submitted to a CO_2_ critical point dryer and coated with gold in a metallizer. The morphology of the fungal hyphae was observed at 10,000× magnification with a high-resolution scanning electron microscope (APREO S, Thermo Scientific, Waltham, MA, USA) operating at an accelerating voltage of 5 kV.

FDA staining assay was used to evaluate the effect of metconazole on the cell viability of *P. oryzae*. A 20-μL droplet conidial suspension (1 × 10^5^ conidia/mL) treated with different concentrations of metconazole was placed on hydrophobic glasses. After 2 h incubation, the conidia samples were stained with FDA solution (10 μg/mL in PBS) for 10 min in darkness and subsequently washed three times with PBS to remove impurities before being observed under a microscope (Olympus BX51, excitation light 545 nm, emission light 590 nm).

### 4.4. Evaluation of Metconazole Activity against Conidial Germination and Germ Tube Elongation

An examination of the inhibitory activity of metconazole against conidial germination and germ tube elongation was conducted under a microscope (Olympus BX51). Briefly, the WT strain P131 was cultured on OTA plates [46] for 7 d to collect conidia. A conidial suspension of 1 × 10^5^ conidia/mL was supplemented with metconazole at a series of concentrations (10, 25 and 50 μg/mL), and a 30 μL droplet of the mixture was incubated on hydrophobic cover glass (48366-067, VWR Corporation, Radnor, PA, USA) at 28 °C under darkness for 2, 8 and 24 h, respectively. Conidial germination rates were counted as the number of germ tubes produced in 50 conidia. Germ tube elongation was determined by measuring the lengths of germ tubes developed from 50 conidia using the ImageJ software (National Institutes of Health, Bethesda, MD, USA).

### 4.5. Evaluation of Metconazole Activity against Appressorium Morphogenesis and Infection Hypae Development in Host Tissues

The effects of metconazole on appressorium formation were analyzed by microscopic observation. 30 μL droplets of the conidial suspension of P131 (1 × 10^5^ conidia/mL) supplemented with metconazole at final concentrations of 0, 10, 25 and 50 μg/mL were inoculated on hydrophobic plastic coverslips (48366-067, VWR Corporation, Radnor, PA, USA), respectively, and incubated at 28 °C under darkness for 8 and 24 h before being photographed using an Olympus BX51 microscope under a bright field. Appressorium formation rate was determined by the number of appressoria produced in 50 germinated conidia.

The effects of metconazole on the infection process of *P. oryzae* were determined on barley leaves. Some equal-volume (10 μL) droplets of conidial suspension (1 × 10^5^ conidia/mL) supplemented with a final concentration of 0, 5, 10 and 25 μg/mL metconazole were placed onto 1-week-old barley leaves (*Hordeum vulgare*) leaves and then incubated in a moist, dark chamber at 28 °C for 32 h. The microscopy observations of invasive hyphal structures were conducted under an Olympus BX51 microscope.

### 4.6. Control Efficacy of Metaconazole to the Virulence of P. oryzae in Barley and Rice

Barley seedlings (*Hordeum vulgare*) grown for one week and rice seedlings (*Oryza sativa* cv. LTH) grown for three weeks as described by Chen et al. [47] were used for virulence tests. Three 10 μL droplets of conidia suspension (1 × 10^5^ conidia/mL) amended with a series of concentraions of metconazole (1, 5, 10, 25 and 50 μg/mL) were spotted onto the barley leaves or wounded rice leaves. All the leaves were incubated under a full-humidity condition at 28 °C with 12 h of darkness and 12 h of light successively in a day. As for the photos, lesion formation was examined at 5 dpi (barley leaves) or 7 dpi (rice leaves) after inoculation. The length of lesions was evaluated using the ImageJ software.

### 4.7. Statistics Analysis

Data were analyzed using the Graphpad prism 8.0 software (GraphPad Software, San Diego, CA, USA). Data are expressed as the mean ± standard deviation (SD). All experiments were repeated three times. The means of the different treatment groups were analyzed using one-way ANOVA with Duncan’s multiple range test to determine the significant differences between the control and the treatment groups. A *p*-value of <0.05 indicates significant differences.

## Data Availability

The data presented in this study are available on request from the corresponding author.

## Supplementary Materials

The following supporting information can be downloaded at: https://www.mdpi.com/article/10.3390/molecules29061353/s1. Figure S1. Metconazole significantly inhibited the mycelial growth of seven *P. oryzae* strains collected from rice paddy fields in Shaoguan city, Guangdong province, China at the concentration of 0.5 μg/mL and 1.0 μg/mL. The statistics of (**a**) colony diameters and (**b**) inhibition rates of the indicated strains. MET: methanol as solvent control. 23-SG-x: strains isolated from paddy fields in Shaoguan city in 2023.
