# Supplementary material for: In Vitro and Ex Vivo Antifungal Activities of Metconazole against the Rice Blast Fungus Pyricularia oryzae
Source: Molecules. 2024 Mar 19;29(6):1353. doi: 10.3390/molecules29061353 (PMC10975861; doi:10.3390/molecules29061353)
Supplement: Supplementary file 1 [file molecules-29-01353-s001.zip › molecules-2891589-supplementary.pdf]

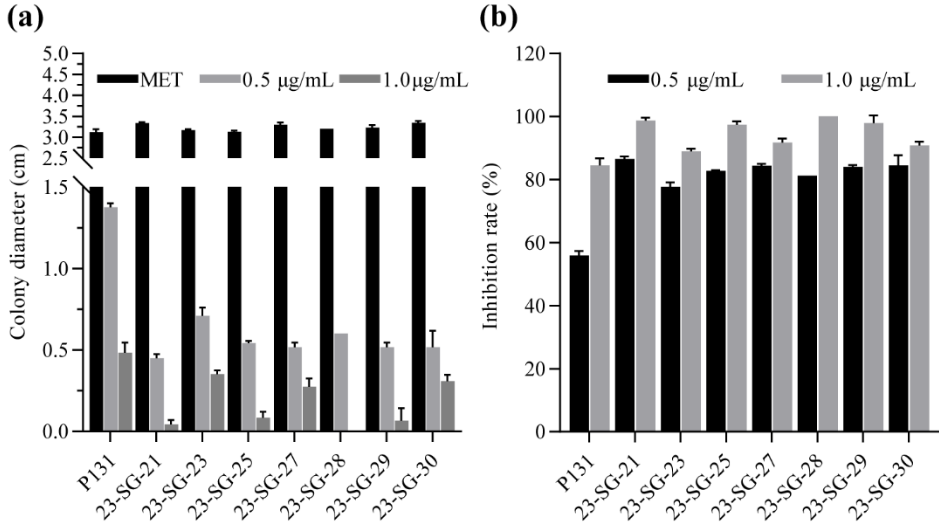

Figure S1. Metconazole significantly inhibited the mycelial growth of P131 and seven *P. oryzae* strains collected from rice paddy fields in Shaoguan city, Guangdong province, China at the concentration of 0.5 μg/mL and 1.0 μg/mL. The statistics of **(a)** colony diameters and **(b)** inhibition rates of the indicated strains. MET: methanol as solvent control. 23-SG-x: strains isolated from paddy fields in Shaoguan city in 2023.
